# Transgenic Expression of *Bmp3b* in Mesenchymal Progenitors Mitigates Age-Related Muscle Mass Loss and Neuromuscular Junction Degeneration

**DOI:** 10.3390/ijms221910246

**Published:** 2021-09-23

**Authors:** Tamaki Kurosawa, Keitaro Minato, Madoka Ikemoto-Uezumi, Jun Hino, Kunihiro Tsuchida, Akiyoshi Uezumi

**Affiliations:** 1 Muscle Aging and Regenerative Medicine, Tokyo Metropolitan Institute of Gerontology, 35-2 Sakae-cho, Itabashi, Tokyo 173-0015, Japan; tamaki-k@g.ecc.u-tokyo.ac.jp (T.K.); kinbensyonen@yahoo.co.jp (K.M.); muezumi@tmig.or.jp (M.I.-U.); 2 Laboratory of Veterinary Pharmacology, Department of Veterinary Medical Sciences, Graduate School of Agriculture and Life Sciences, Tokyo University, 1-1-1 Yayoi, Bunkyo-ku, Tokyo 113-8657, Japan; 3 Department of Regenerative and Transplant Medicine, Division of Orthopedic Surgery, Graduate School of Medical and Dental Sciences, Niigata University, 1-757 Asahimachi-Dori, Tyuo-Ku, Niigata 951-8510, Japan; 4 Department of Biochemistry, National Cerebral and Cardiovascular Center Research Institute, 6-1 Kishibe-Shimmachi, Suita 564-8565, Japan; jhino@ncvc.go.jp; 5 Division for Therapies against Intractable Diseases, Institute for Comprehensive Medical Science, Fujita Health University, 1-98 Dengakugakubo, Kutsukake-cho, Toyoake 470-1192, Japan; tsuchida@fujita-hu.ac.jp

**Keywords:** skeletal muscle, sarcopenia, mesenchymal progenitors

## Abstract

Skeletal muscle is a vital organ for a healthy life, but its mass and function decline with aging, resulting in a condition termed sarcopenia. The etiology of sarcopenia remains unclear. We recently demonstrated that interstitial mesenchymal progenitors are essential for homeostatic muscle maintenance, and a diminished expression of the mesenchymal-specific gene *Bmp3b* is associated with sarcopenia. Here, we assessed the protective function of *Bmp3b* against sarcopenia by generating conditional transgenic (Tg) mice that enable a forced expression of *Bmp3b* specifically in mesenchymal progenitors. The mice were grown until they reached the geriatric stage, and the age-related muscle phenotypes were examined. The Tg mice had significantly heavier muscles compared to control mice, and the type IIB myofiber cross-sectional areas were preserved in Tg mice. The composition of the myofiber types did not differ between the genotypes. The Tg mice showed a decreasing trend of fibrosis, but the degree of fat infiltration was as low as that in the control mice. Finally, we observed the preservation of innervated neuromuscular junctions (NMJs) in the Tg muscle in contrast to the control muscle, where the NMJ degeneration was conspicuous. Thus, our results indicate that the transgenic expression of *Bmp3b* in mesenchymal progenitors alleviates age-related muscle deterioration. Collectively, this study strengthens the beneficial role of mesenchymal *Bmp3b* against sarcopenia and suggests that preserving the youthfulness of mesenchymal progenitors may be an effective means of combating sarcopenia.

## 1. Introduction

The skeletal muscle is the largest organ in the human body. The skeletal muscle is an essential component of the musculoskeletal system and is essential for exercise and physical activity. The skeletal muscle also has a significant impact on the whole-body metabolism. It is the largest target organ of insulin and serves as a major reservoir of amino acids. Thus, the skeletal muscle is vital for a healthy life. However, skeletal muscle mass and function gradually decline with age, eventually leading to a pathological condition termed sarcopenia. Sarcopenia not only reduces physical activity but also deteriorates the general health and is therefore one of the major determinants of a healthy life expectancy. A recent epidemiological study confirmed that sarcopenia is strongly associated with an increased risk of all-cause mortality and disability [1]. Since the population is rapidly aging globally, preventing sarcopenia is an important task for public health.

Multiple factors are involved in the etiology of sarcopenia. Due to its usefulness in clinical practice, sarcopenia is divided into two categories: primary and secondary sarcopenia [2]. Primary sarcopenia is a condition in which no other cause is evident except for aging, and secondary sarcopenia is a condition in which one or more other causes are evident. From a cellular perspective, the involvement of muscle stem cells in sarcopenia has attracted growing attention, as the skeletal muscle is a typical tissue with high regenerative potential that is affected by aging. The regeneration of skeletal muscle depends completely on tissue-specific stem cells called satellite cells. The satellite cell regenerative function was reported to decline in geriatric mice (>28 months old) but not in old mice (20–24 months old) [3]. However, sarcopenic symptoms, such as decreased muscle mass and function, become apparent before the geriatric phase and are already evident in the old phase where the satellite cell function is well-preserved [4,5,6,7]. Furthermore, genetically engineered mice with conditional satellite cell depletion showed an almost normal muscle phenotype with no exacerbation of sarcopenia [8,9]. Therefore, impairment in the satellite cell function is not related to the onset of primary sarcopenia, although it might be involved in injury-related secondary sarcopenia.

Mesenchymal progenitors represent another progenitor population residing in the skeletal muscle [10]. They are also known as fibrogenic/adipogenic progenitors [11]. Mesenchymal progenitors, which specifically express platelet-derived growth factor receptor α (PDGFRα), have been demonstrated to be the origin of ectopic fat cells in muscle [10,11]. Since ectopic adipogenesis in muscle occurs in aging muscle [12], mesenchymal progenitors are thought to be involved in the process of sarcopenia. Recently, Wosczyna et al. and our group revealed the essential role of mesenchymal progenitors in steady-state muscle maintenance [13,14]. Mesenchymal progenitors reside in the muscle interstitium, and some of them lie adjacent to motor nerve axons and cover the neuromuscular junctions (NMJs). The genetic ablation of these cells resulted in myofiber atrophy and partial or complete denervation at the NMJs [13,14], leading to a new concept that mesenchymal progenitors are required for the maintenance of NMJs, as well as muscle mass [15]. We further explored the mechanism by which mesenchymal progenitors maintain muscle integrity and identified the mesenchymal progenitor-specific gene *Bmp3b* [13]. *Bmp3b* is highly expressed in mesenchymal progenitors residing in young muscle, but its expression level is significantly decreased by aging [13]. Using *Bmp3b*-deficient mice and cultured cells, we demonstrated that *Bmp3b* has pleiotropic effects on muscle myofibers and NMJs by stimulating hypertrophic signaling pathways and Schwann cell characteristics, which positively influence the muscle mass and NMJ stability [13]. Therefore, age-related changes in mesenchymal progenitors significantly affect the development of sarcopenia, and *Bmp3b* represents a trophic factor that assumes mesenchymal progenitor-dependent muscle maintenance.

In this study, we developed conditional transgenic (Tg) mice that enabled a forced expression of *Bmp3b* specifically in mesenchymal progenitors to further explore the function of *Bmp3b* in muscle aging. The Tg mice were grown until they reached the geriatric stage, and their muscle phenotypes were analyzed. Some age-related muscle deteriorations were alleviated in the Tg mice compared to their wild-type (WT) littermates. Our results confirmed the protective action of *Bmp3b* against sarcopenia and suggested that preserving the quality of mesenchymal progenitors can be a promising strategy to combat sarcopenia.

## 2. Results

**Figure 1:**
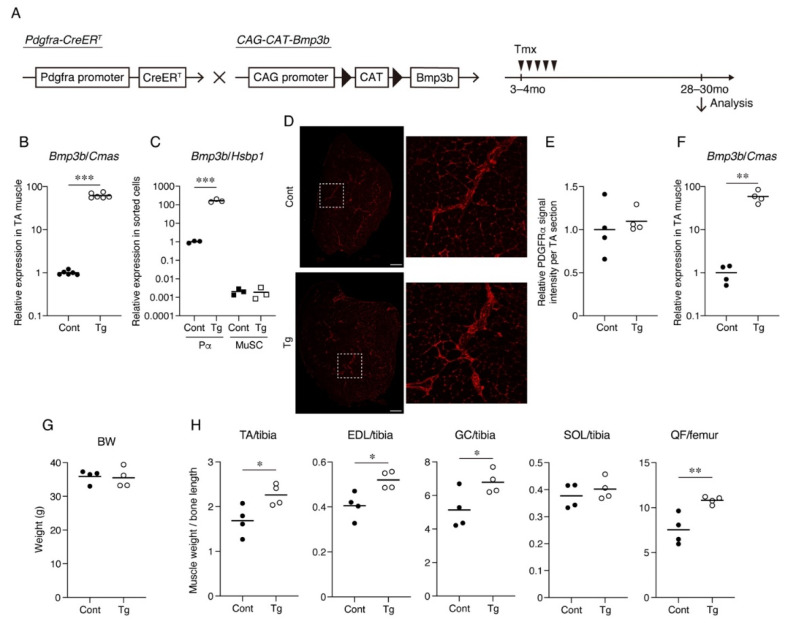
Mesenchymal *Bmp3b* Tg mice exhibit heavier muscle weights. (**A**) Mesenchymal *Bmp3b* Tg mice were generated by crossing *Pdgfra-CreER* mice and *CAG-CAT-Bmp3b* mice. In *Pdgfra-CreER*/*CAG-CAT-Bmp3b* mice, the administration of tamoxifen allows the translocation of CreER to the nucleus and excision of the floxed *CAT* gene, resulting in the expression of *Bmp3b* under the control of the CAG promoter specifically in *Pdgfra*^+^ mesenchymal progenitors. (**B**) The expression of *Bmp3b* in the tibialis anterior (TA) muscle was compared between the Tg mice and littermate control mice (Cont, *WT*/*Pdgfra-CreER*, or *WT*/*CAG-CAT-Bmp3b*). (**C**) The expression of *Bmp3b* in sorted PDGFRα^+^ cells (Pα) or satellite cells (MuSC) was compared between the Tg mice and littermate control mice (Cont, *WT*/*CAG-CAT-Bmp3b*). (**D**) TA muscle sections of the Tg mice and littermate control mice (Cont, *WT*/*CAG-CAT-Bmp3b*) were stained with an antibody against PDGFRα (red). The right panels are magnified views of the boxed regions in the left panels. (**E**) The PDGFRα signal intensity per TA section was quantified. (**F**) The expression of *Bmp3b* in the TA muscle was compared between the geriatric Tg mice and geriatric littermate control mice (Cont, *WT*/*CAG-CAT-Bmp3b*). (**G**) The body weights were comparable between the genotypes. (**H**) The TA, extensor digitorum longus (EDL), gastrocnemius (GC), soleus (SOL), and quadriceps femoris (QF) weights were measured and then normalized by the bone length. The normalized muscle weight was compared between the Tg mice and littermate control mice (Cont, *WT*/*CAG-CAT-Bmp3b*). The data are expressed as the means and individual data points; two-sided unpaired *t*-test. *n* = 6 mice (**B**), *n* = 3 mice (**C**), and *n* = 4 mice per genotype (**E**–**H**). * *p* < 0.05, ** *p* < 0.01, and *** *p* < 0.001. Scale bar: 300 μm (**D**).

*Bmp3b* is specifically expressed in mesenchymal progenitors, but its expression level is significantly decreased by aging [13]. Additionally, *Bmp3b* deficiency leads to a loss of muscle mass and degeneration of NMJs [13]. Thus, we sought to clarify whether a sustained *Bmp3b* expression can prevent the muscle deterioration caused by aging. To induce the expression of the *Bmp3b* transgene in mesenchymal progenitors, *Pdgfra-CreER* mice were crossed with *CAG-CAT-Bmp3b* mice (Figure 1A). In *CAG-CAT-Bmp3b* mice, the induction of Cre-mediated recombination allows the excision of the floxed *CAT* gene, leading to the expression of *Bmp3b* under the control of the CAG promoter specifically in Cre-expressing cells [16]. A specific Cre-mediated recombination in mesenchymal progenitors using *Pdgfra-CreER* mice was demonstrated in our previous study [13]. Hereafter, *Pdgfra-CreER*/*CAG-CAT-Bmp3b* mice are referred to as Tg mice. In this study, the Tg mice and their wild-type (WT) littermates were administered the same amount of Tmx to exclude the nonspecific effects of Tmx. We confirmed a significant upregulation of *Bmp3b* in the skeletal muscle of the Tg mice (Figure 1B). We also confirmed a significant upregulation of *Bmp3b* in the sorted PDGFRα^+^ cells of the Tg mice but not in the satellite cells (Figure 1C). Having confirmed the induction of the transgene, the mice assigned to the aging cohort were grown until they reached the geriatric stage (28–30 months old). Only four mice survived in each genotype, and they were subjected to various analyses. PDGFRα immunostaining revealed that PDGFRα^+^ cells were frequently observed in the interstitial space in both genotypes (Figure 1D,E). Thus, the mesenchymal progenitors appeared to be preserved at a similar level in both the Tg and control muscles. When compared with the control mice, the geriatric Tg mice showed much higher levels of *Bmp3b* expression (Figure 1F), indicating that the transgenic expression of *Bmp3b* was maintained during the aging of the Tg mice. Although the body weight did not differ significantly, the hind limb muscles of the Tg mice were significantly heavier than those of the control (Cont, *WT*/*CAG-CAT-Bmp3b*) mice (Figure 1G,H). One exception was the soleus muscle, which showed no difference between the Tg and control mice (Figure 1H). This may be because slow-twitch muscles are relatively resistant to age-related atrophy compared to fast-twitch muscles [17,18].

**Figure 2:**
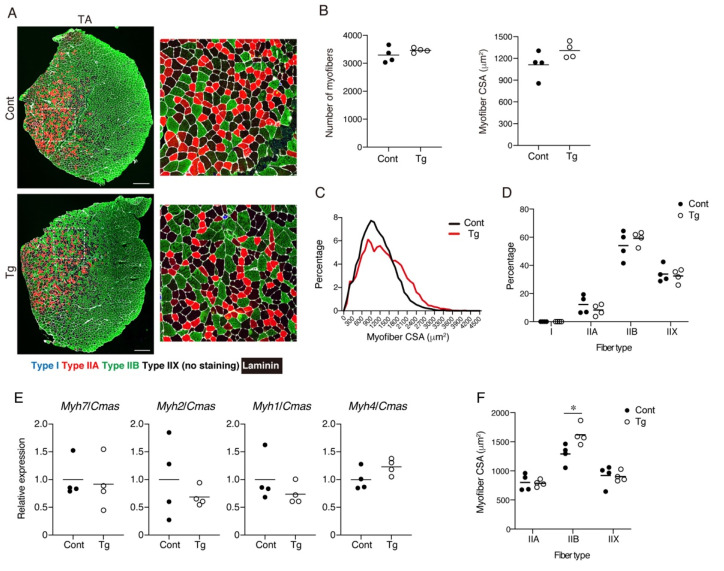
An analysis for the number, cross-sectional areas, and subtypes of the myofibers. (**A**) Tibialis anterior (TA) muscle sections of the Tg mice and littermate control mice (Cont, *WT*/*CAG-CAT-Bmp3b*) were stained with antibodies against MyHC I (blue), MyHC IIA (red), MyHC IIB (green), and laminin (white). The right panels are magnified views of the boxed regions in the left panels. (**B**) The number and cross-sectional areas (CSA) of the myofibers are shown. (**C**) The CSA distribution is displayed by a histogram. Note that the myofibers of the Tg mice are distributed in the larger region, while those of the control mice are distributed in the smaller region. (**D**) The percentage of each fiber type is shown. (**E**) The gene expression levels for *Myh7* (type I), *Myh2* (type IIA), *Myh1* (type IIX), and *Myh4* (type IIB) are shown. (**F**) The CSA of each fiber type was measured. The data are expressed as the means and individual data points; two-sided unpaired *t*-test. *n* = 4 mice per genotype. * *p* < 0.05. Scale bar: 300 μm (**A**).

The myofiber number, CSA, and muscle fiber type were analyzed by immunohistochemical staining. The TA muscle was stained for myosin heavy-chain (MyHC) type I, MyHC type IIA, MyHC type IIB, and laminin (Figure 2A). This staining procedure enabled the detection of type I^+^, type IIA^+^, and type IIB^+^ myofibers, and the myofibers negative for these three types of MyHC were considered as type IIX myofibers (Supplemental Material Figure S1) [19]. The number of myofibers did not significantly differ between the genotypes, but the myofiber CSA tended to be larger in the Tg mice, although the difference was not statistically significant (Figure 2B). When the distribution of myofiber CSA was displayed on a histogram plot, the Tg mice demonstrated larger myofibers compared to the control mice (Figure 2C). No statistically significant difference was observed in the percentages of the muscle fiber types (Figure 2D). The gene expression levels for the four myosin heavy chains also did not significantly differ between the genotypes (Figure 2E). When the CSA of each fiber type was measured, the type IIB myofiber CSA of the Tg mice was significantly larger than that of the control mice (Figure 2F), indicating that the type IIB myofibers were protected from age-related atrophy by mesenchymal *Bmp3b* overexpression.

**Figure 3:**
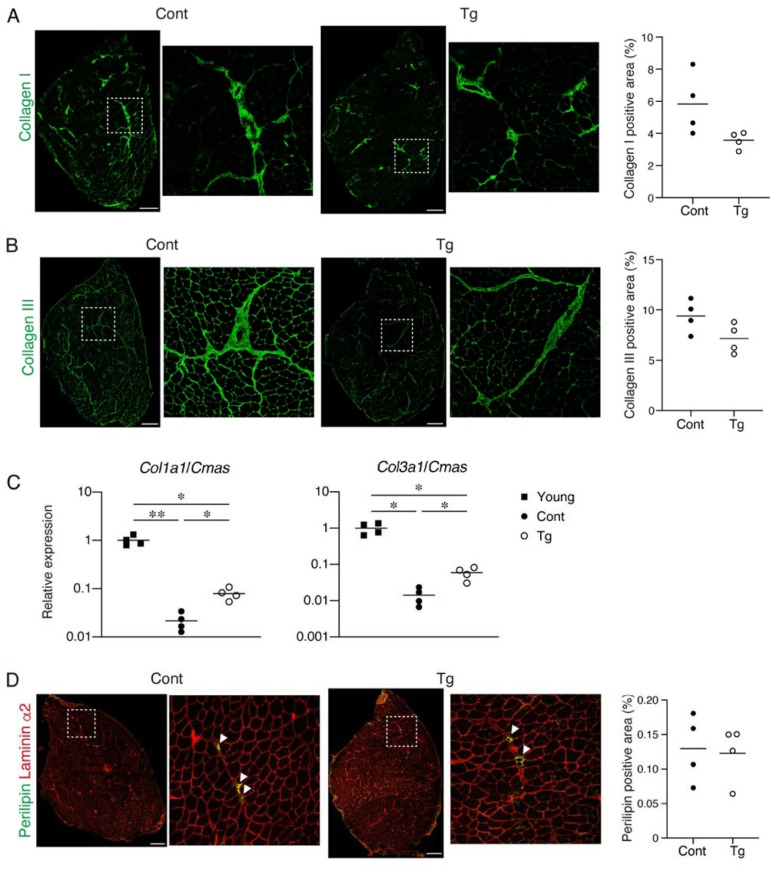
The extent of the fibrosis and fat infiltration in the skeletal muscle. (**A**) Tibialis anterior (TA) muscle sections of the Tg mice and littermate control mice (Cont, *WT*/*CAG-CAT-Bmp3b*) were stained with an antibody against collagen I (green). The right panels are magnified views of the boxed regions in the left panels. The percentage of the collagen I^+^ area is shown. (**B**) The TA muscle sections of the Tg mice and littermate control mice (Cont, *WT*/*CAG-CAT-Bmp3b*) were stained with an antibody against collagen III (green). The right panels are magnified views of the boxed regions in the left panels. The percentage of the collagen III^+^ area is shown. (**C**) The gene expression levels of *Col1a1* and *Col3a1* in the young, geriatric control (Cont, *WT*/*CAG-CAT-Bmp3b*) and the geriatric Tg mice are shown. (**D**) The TA muscle sections of the Tg mice and littermate control mice (Cont, *WT*/*CAG-CAT-Bmp3b*) were stained with antibodies against perilipin (green) and laminin α2 (red). The right panels are magnified views of the boxed regions in the left panels. The arrowheads indicate perilipin^+^ ectopic adipocytes located in the interstitial space. The percentage of the perilipin^+^ area is shown. The data are expressed as the means and individual data points; two-sided unpaired *t*-test (**A**,**B**,**D**). Brown–Forsythe and Welch ANOVA test (**C**). *n* = 4 mice. * *p* < 0.05 and ** *p* < 0.01. Scale bar: 300 μm.

Since increased fibrosis and fat infiltration are hallmarks of aged skeletal muscle [12,20], we next examined these pathological changes. We observed a trend for lower collagen I^+^ and collagen III^+^ interstitial areas in the muscles of the Tg mice, suggesting that the Tg mice developed less fibrosis compared to the control mice (Figure 3A,B). It has been demonstrated that the mRNA levels for collagens decrease, while the accumulation of collagen proteins increases with aging [21,22,23]. Therefore, the increased muscle fibrosis with age is not the result of increased collagen gene expression but is most likely due to a decreased degradation capacity that leads to an impaired collagen turnover. We confirmed that the gene expression levels of *Col1a1* and *Col3a1* were significantly lower in the geriatric control mice compared with the young mice (Figure 3C). However, the reduction in the collagen gene expression was attenuated in the geriatric Tg mice (Figure 3C), suggesting that the transgenic expression of *Bmp3b* inhibits the age-related impairment of collagen turnover. Perilipin-positive ectopic adipocytes were rare in the muscles of both genotypes, and there was no difference in the fat infiltration between the genotypes (Figure 3B). This result is because mice rarely develop ectopic adipocytes, even in old age, under noninjured conditions [24].

**Figure 4:**
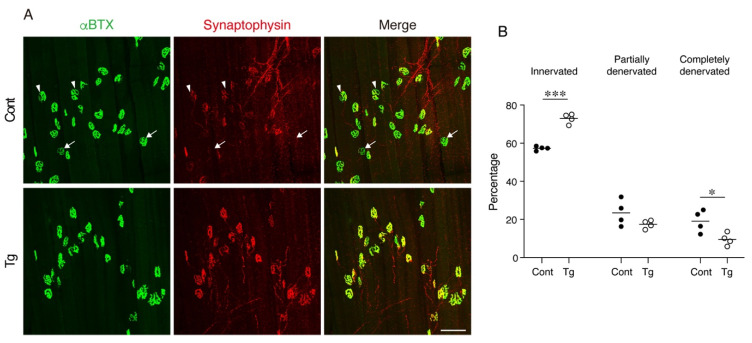
Mesenchymal transgenic expression of Bmp3b preserves the neuromuscular junction from age-related degeneration. (**A**) Whole-mount immunofluorescence staining of the extensor digitorum longus (EDL) muscles of the Tg mice and littermate control mice (Cont, *WT*/*CAG-CAT-Bmp3b*) for the acetylcholine receptor (α-bungarotoxin: αBTX, green) and synaptophysin (red). The arrowheads and arrows indicate partially and completely denervated neuromuscular junctions (NMJs), respectively. (**B**) The ratios of the innervated, partially denervated, and completely denervated NMJ per mice were calculated. The data are expressed as the means and individual data points; two-sided unpaired *t*-test. *n* = 4 mice per genotype. * *p* < 0.05 and *** *p* < 0.001. Scale bar: 100 μm.

The degeneration of NMJ is a typical age-related deterioration of the skeletal muscle. We investigated the status of individual NMJ by a staining-based methodology that was shown to detect age-related NMJ degeneration efficiently [25]. In normal healthy NMJs, α-bungarotoxin-stained postsynaptic endplate regions are completely covered with synaptophysin-positive presynaptic nerve terminals. However, aging increases the ratio of degenerated NMJs, where the postsynaptic regions partially or completely lose their counterpart presynaptic nerve terminals. NMJ degeneration is prominent in fast-twitch extensor digitorum longus (EDL) but not in the slow-twitch soleus muscles of geriatric mice [25]. Therefore, we examined the NMJs in the EDL muscle by whole-mount immunofluorescence staining. In control geriatric mice, we frequently observed partially or completely denervated NMJs (Figure 4A,B). In contrast, the Tg mice showed less completely denervated NMJs and a trend for fewer partially denervated NMJs, resulting in more innervated NMJs compared to the control mice (Figure 4A,B). These results indicate that the transgenic expression of *Bmp3b* in mesenchymal progenitors preserves the NMJ from age-related deterioration and, thus, maintains the skeletal muscle health during aging.

## 3. Discussion

Age-related changes in myofibers have been extensively studied to determine the cause of sarcopenia, because the skeletal muscle is mainly composed of myofibers. However, the etiology of sarcopenia remains largely elusive. During the development of sarcopenia, the loss of muscle strength occurs more rapidly than the loss of muscle mass [26]. Studies measuring both in vivo muscle strength and in vitro myofiber contractile properties demonstrated that, although the in vivo muscle strength is compromised by aging and further exacerbated by disuse, the contractile force generated by single myofibers in vitro is unaffected even in the non-ambulatory oldest old subjects [27,28]. These results strongly suggest that the intrinsic myofiber function is preserved during aging and that factors other than myofibers, such as age-related changes in the neuromuscular or extracellular matrix components, are responsible for the development of sarcopenia. Therefore, studying non-myofiber components in the skeletal muscle is important for a better understanding of the mechanisms of sarcopenia.

Mesenchymal progenitors may represent an important non-myofiber component in the development of sarcopenia. These cells were first identified as progenitors that generate ectopic adipocytes in skeletal muscles [10,11]. Subsequently, they have also been demonstrated to be the origin of fibrosis and heterotopic ossification [29,30]. These pathological features of mesenchymal progenitors have been well-recognized and investigated in many studies, but the role of these progenitors in normal healthy conditions remains unexplored. To this end, Wosczyna et al. generated mice that enable the conditional depletion of cells expressing PDGFRα, a specific marker of mesenchymal progenitors [10], and found that the depletion of mesenchymal progenitors results in muscle atrophy [14]. We also generated PDGFRα^+^ cell-depleted mice and demonstrated that mesenchymal progenitor depletion leads to phenotypes markedly similar to sarcopenia, including a loss of muscle mass and strength, myofiber atrophy, and degeneration of neuromuscular junctions [13]. These studies clearly indicated that mesenchymal progenitors play a critical role in maintaining the integrity of the skeletal muscle tissue.

To clarify the involvement of mesenchymal progenitors in the development of sarcopenia, we explored age-related changes in mesenchymal progenitors and found a significant reduction in the expression of the mesenchymal progenitor-specific gene *Bmp3b* [13]. *Bmp3b*-deficient mice exhibited atrophied myofibers, reduced muscle strength, and NMJ degeneration, indicating that *Bmp3b* is functionally important for the maintenance of NMJs, in addition to muscle mass and strength [13]. Therefore, the *Bmp3b* protein confers a supportive function to mesenchymal progenitors. In this study, to strengthen the importance of *Bmp3b* in the maintenance of skeletal muscle health, *Bmp3b* was specifically overexpressed in mesenchymal progenitors, and its effect on muscle aging was investigated. In contrast to the phenotypes observed in *Bmp3b*-deficient mice, the mesenchymal-specific *Bmp3b* Tg mice showed preserved type IIB myofiber mass and NMJs against aging. The selective protection of type IIB fibers further supports the beneficial effect of *Bmp3b* against aging, because type II fiber atrophy, especially type IIB fiber atrophy, dominantly contributes to sarcopenia [19,31]. The transgenic expression of *Bmp3b* seems to exert a positive influence directly on myofibers, as we have previously shown that recombinant *Bmp3b* stimulates hypertrophic signals such as Akt and Smad-1/-5/-8 in differentiated muscle cells [13]. We have also shown that recombinant *Bmp3b* positively regulates the characteristics of cultured Schwann cells by stabilizing the differentiated states [13]. Schwann cells are important for the formation and maintenance of the NMJs [32], and the age-related denervation of NMJs is accompanied by the degeneration of Schwann cells [25]. Thus, the transgenic expression of *Bmp3b* presumably preserves NMJs by stabilizing Schwann cells. Since the expression levels of *Bmp3b* are significantly decreased by aging, maintaining the youthfulness of mesenchymal progenitors would be an effective means for preventing sarcopenia.

We used *Pdgfra-CreER* mice to induce a transgene expression in mesenchymal progenitors, because PDGFRα is specifically expressed by mesenchymal progenitors within the skeletal muscle [10]. However, PDGFRα is also expressed by similar mesenchymal cells in other tissues, leading to the induction of *Bmp3b* in PDGFRα^+^ cells residing outside of the muscle in our Tg mice. Thus, we cannot exclude the effect on the Tg mouse phenotype of *Bmp3b* produced by PDGFRα^+^ cells residing in other tissues. However, BMPs have been demonstrated to act mainly through the local paracrine action. It is well-known that TGF-β superfamily members, especially BMPs, act in the restricted region during development. Some BMPs have been reported to influence and induce ventral fates only in the regions in which they are expressed, and the action of the BMPs is tightly restricted to the regions within and around the cells that produce them [33]. Given the reported paracrine action of BMPs, Bmp3b produced from muscle-resident mesenchymal progenitors may be responsible for the phenotypes observed in our Tg mice.

Although this study focused on the roles of mesenchymal progenitors in a steady-state condition, recent studies uncovered molecular mechanisms through which mesenchymal progenitors support muscle regeneration. Mesenchymal progenitors secrete WNT1 Inducible Signaling Pathway Protein 1 (WISP1), which is required for efficient muscle regeneration [34]. Aged mesenchymal progenitors produce less WISP1, leading to the impaired myogenic commitment of muscle stem cells [34]. The WNT5a/GSK3/β-catenin axis inhibits the adipogenic differentiation of mesenchymal progenitors and stimulates the production of follistatin, which is responsible for the promyogenic activity of mesenchymal progenitors [35].

An increased fibrosis and fat cell infiltration are typical age-related changes in the skeletal muscle [12,20]. Mesenchymal progenitors contribute to these pathological changes by differentiating them into fibroblasts and adipocytes. Thus, in this study, the effect of *Bmp3b* overexpression on age-related fibrosis and fat infiltration in skeletal muscle was examined. We observed a decreased tendency of fibrosis but no significant change in the fat infiltration in the mesenchymal-specific *Bmp3b* Tg muscle. This is probably because aged mice rarely develop ectopic adipogenesis at the baseline condition [24]. Moreover, the geriatric control mice exhibited few ectopic adipocytes. Thus, the mouse model may not be suitable for studying fat infiltration in the skeletal muscle caused by aging. However, a recent study reported an antiadipogenic effect of *Bmp3b* on mesenchymal progenitors in an injured muscle [36]. It was shown that *Bmp3b* secreted from the CD142^+^ subpopulation of mesenchymal progenitors suppresses the adipogenic differentiation of CD142^-^ cells predisposed to adipogenesis [36]. The antiadipogenic property of *Bmp3b* further reinforces the importance of this factor in maintaining muscle health. In addition, studies on adipose tissue have revealed the protective role of *Bmp3b* against obesity and the metabolic syndrome [16,37]. Therefore, we believe that *Bmp3b* prevents sarcopenia by preserving the muscle muss and NMJs and suppressing the aberrant differentiation of the mesenchymal progenitors.

Some of the analyses in this study (Figure 2B and Figure 3A,B) could not reach statistical significance. We originally prepared 15 control and 14 Tg mice for the aging cohort. However, only four mice survived in each genotype, which made some of the analyses underpowered. Therefore, more mice will be required to possibly show a statistical significance in these assays. Using the current data, the effect sizes for Figure 2B and Figure 3A,B were estimated as 1.29, 1.6, and 1.45, respectively. Accordingly, the sample sizes required to show statistical significance in Figure 2B and Figure 3A,B were determined as 11, 8, and 9, respectively, for a type I error rate = 0.05 and power = 0.8. Therefore, the originally assigned number of mice was appropriate, but enough number of mice did not survive in this study. Although we obtained only four geriatric mice in each genotype, the muscle weight and innervation status of the NMJs were preserved in the Tg mice with statistically significant levels, suggesting the beneficial effects of sustained *Bmp3b* expression against aging. Another limitation of this study was the lack of a physiological assessment. Since this study mainly depended on histological and gene expression analyses, the muscle functions should have been evaluated to further establish the beneficial role of mesenchymal progenitor-derived *Bmp3b* in ameliorating sarcopenia.

Taken together, we demonstrated the anti-sarcopenic effects of *Bmp3b* using mesenchymal-specific *Bmp3b* Tg mice. Although our results reinforced the importance of *Bmp3b*, mesenchymal progenitors should express other factors that play a role in maintaining the muscle integrity in addition to *Bmp3b*. Therefore, inhibiting the age-related deterioration of mesenchymal progenitors, including a diminished expression of *Bmp3b*, may represent an effective therapeutic strategy for the prevention of sarcopenia.

## 4. Materials and Methods

### 4.1. Mice

*Pdgfra-CreER* mice [38] (stock #018280, The Jackson Laboratory, Bar Harbor, ME, USA) were purchased from The Jackson Laboratory. *CAG-CAT-mBmp3b* Tg mice have been previously described [16]. The genetically engineered mice were backcrossed with C57BL/6 mice at least six times. Three- to four-month-old mice were intraperitoneally injected with tamoxifen (Tmx, 4 mg) for five consecutive days to induce a recombination. Nine-week-old male C57BL/6 mice were used as the young mice.

### 4.2. RNA Extraction and Quantitative RT-PCR

The total RNA was extracted from the muscle tissues using the miRNeasy Mini Kit (Qiagen, Hilden, Germany), and equal amounts of RNA were reverse-transcribed into cDNA using the QuantiTect Reverse Transcription Kit (Qiagen). Real-time quantitative PCR was performed with TB Green Premix Ex Taq II (TaKaRa, Shiga, Japan) using a Thermal Cycler Dice Real-Time System (TaKaRa) under the following cycling conditions: 94 °C for 30 s, followed by 40 cycles of amplification (94 °C for 5 s, 60 °C for 20 s, and 72 °C for 10 s) and a dissociation curve analysis. *Cytidine monophospho-N-acetylneuraminic acid synthetase* (*Cmas*) [39,40] and *heat shock factor-binding protein 1* (*Hsbp1*) [41] were shown to be suitable as the internal control genes for the muscle tissue and sorted cells, respectively. The primer sequences were 5′-CTTTGACGCCTACTACTGTGCTG-3′ and 5′-AAGGGAGTTCATCTTGTCTGGAA-3′ for *Bmp3b* (product size, 157 bp), 5′-CCGAACCCCAAGGAAAAGAA-3′ and 5′-GTGGACATTAGGCGCAGGAA-3′ for *Col1a1* (product size, 134 bp), 5′-CTCAAATGGCTCACCAGGAC-3′ and 5′-CACCAGGACTGCCGTTATTC-3′ for *Col3a1* (product size, 101 bp), 5′-GGAGGACCAAGTGAGTGAGC-3′ and 5′-TCGTCTAGCTGGCGTGAGTA-3′ for *Myh1* (product size, 123 bp), 5′-AGCGACTGATCAACGACCTG-3′ and 5′-AACTGAGATACCAGCGCTTCC-3′ for *Myh2* (product size, 103 bp), 5′-AAACCACCTCAGAGTTGTGGA-3′ and 5′-GTTCCGAAGGTTCCTGATTGC-3′ for *Myh4* (product size, 172 bp), 5′-GGCAAGGCAAAGAAAGGCTC-3′ and 5′-GTTGTCCATCACCCCTGGAG-3′ for *Myh7* (product size, 153 bp), 5′-CAAAGGCATCCCACTGAAGA-3′ and 5′-CCCACACACTCTGGAAGACC-3′ for *Cmas* (product size, 104 bp), and 5′-CAAGACCATGCAGGACATCAC-3′ and 5′-AGGTCAGCGATATTCTTCTCCA-3′ for *Hsbp1* (product size, 147 bp).

### 4.3. Immunofluorescent Staining and Microscopy

Fresh muscle samples were rapidly frozen in isopentane cooled with liquid nitrogen. For staining the subtypes of myosin heavy chain (MyHC), fresh-frozen sections were fixed with acetone for 5 min at −20 °C and blocked using a Mouse-on-Mouse detection kit (Vector, Burlingame, CA, USA). In other cases, fresh-frozen sections were fixed with 4% PFA for 5 min and blocked with a protein block serum-free reagent (Agilent, Santa Clara, CA, USA) for 15 min. The specimens were incubated with the primary antibodies at 4 °C overnight, followed by secondary staining. The primary and secondary antibodies used were anti-PDGFRα (2.5 μg/mL; R&D, Minneapolis, MN, USA, #AF1062), anti-MyHC I (1:10; DSMZ, Braunschweig, Germany, clone: BA-F8), anti-MyHC IIA (1:10; DSMZ, clone: SC-71), anti-MyHC IIB (1:10; DSMZ, clone: BF-F3), anti-laminin (1:400; Sigma-Aldrich, St. Louis, MO, USA, #L9393), anti-collagen I (1:200; Abcam, Cambridge, UK, #ab21286), anti-collagen III (1:100; Abcam, #ab7778), anti-perilipin (1:200, Sigma-Aldrich, #P1873), anti-laminin α2 (1:200; Santa Cruz Biotechnology, Dallas, TX, USA, #sc-59854), Dylight 405 anti-mouse IgG2b (1:1000; Jackson ImmunoResearch, West Grove, PA, USA, #115-475-207), Alexa Fluor 555 anti-mouse IgG1 (1:1000; Thermo Fisher, Waltham, MA, USA, #A21127), Alexa Fluor 647 anti-mouse IgM (1:1000; Thermo Fisher, #A21238), Alexa Fluor 488 anti-rabbit IgG (1:1000; Thermo Fisher, #A32790), and Alexa Fluor 594 anti-rat IgG (1:1000; Thermo Fisher, #A11007). The stained samples were counterstained with DAPI (Dojindo, Kumamoto, Japan) and mounted with SlowFade Gold antifade reagent (Thermo Fisher). The immunofluorescent images were obtained using the inverted fluorescence microscope DMI6000B (Leica, Wetzlar, Germany), BZ-X710 (Keyence, Osaka, Japan), and confocal laser scanning microscope system TCS SP8 (Leica).

### 4.4. Quantitative Analysis of Myofibers

A quantitative analysis of the myofibers was performed as previously described [13]. Briefly, cross-sections were obtained by cutting at the mid-belly of the muscle (approximately 3 mm from the proximal end of the tibialis anterior (TA) muscle). Fluorescent images of the entire cross-sections were captured using the fluorescent microscope system BZ-X710 (Keyence). The images were recognized and quantified using the Hybrid Cell Count application (ver. 1.3.1.1, Keyence). Laminin-stained basal lamina was first recognized based on the intensity of the fluorescent signal by adjusting the threshold, and the myofibers were recognized using an inversion function. The separation function was used to efficiently separate the individual myofibers. Small, misrecognized areas were excluded by adjusting the lower limit of the histogram function. Finally, the errors in the recognition step were manually corrected. The myofiber numbers and cross-sectional areas (CSA) of the individual myofibers were subsequently calculated. The percentage of each type of myofiber was calculated by first detecting the total number of myofibers as described above and then detecting the MyHC I-, IIA-, or IIB-stained fibers using the mask function of the Hybrid Cell Count application (Keyence). The myofibers negative for MyHC I, IIA, and IIB were considered as type IIX myofibers. The collagen I^+^, III^+^, or perilipin^+^ areas were measured using the Hybrid Cell Count application (Keyence).

### 4.5. Whole-Mount Immunofluorescence Staining

The extensor digitorum longus (EDL) muscle was split into four pieces and fixed with 4% PFA for 30 min. After washing with phosphate-buffered saline (PBS), the muscles were blocked by incubating overnight in a blocking solution consisting of 1% Triton X-100 and 4% BSA in PBS at 4 °C, followed by incubation for 1 day with anti-synaptophysin antibody (1:200; Abcam, #ab14692) diluted with the blocking solution at 4 °C with rotation. Subsequently, the muscles were incubated for 1 day with Alexa Fluor 488 α-Bungarotoxin (1:1000; Thermo Fisher, #1313422) and Alexa Fluor 594 anti-rabbit IgG (1:1000; Jackson ImmunoResearch, #711-585-152) diluted with the blocking solution at 4 °C with rotation. The stained muscles were counterstained with DAPI (Dojindo) and mounted with SlowFade Gold antifade reagent (Thermo Fisher). Z-stack images were captured using the confocal laser scanning microscope system TCS SP8 (Leica).

### 4.6. Assessment of NMJ Status

The maximum intensity projection images were reconstructed from Z-stack images obtained from whole-mount EDL staining using LAS X software (ver. 3.5.5.19976, Leica). For the NMJ assessment, we analyzed at least 100 NMJs per mouse. The number of completely denervated, partially denervated, and innervated NMJs were counted.

### 4.7. Statistical Analysis

All the quantitative analyses were performed in a blinding manner. Statistical significance was assessed using GraphPad Prism 8 (ver. 8.4.1, GraphPad Software, San Diego, CA, USA). For the test for normal distribution, Shapiro-Wilk test was used. For comparisons between two groups, a two-tailed unpaired Student’s *t*-test was used. For comparisons of more than two groups, Brown–Forsythe and a Welch one-way analysis of variance (ANOVA) followed by Dunnett’s T3 multiple comparisons test were used. The statistical significance was set at *p* < 0.05.

## Data Availability

Not applicable.

## Supplementary Materials

The following are available online at https://www.mdpi.com/article/10.3390/ijms221910246/s1.
