# Supplementary material for: Transgenic Expression of Bmp3b in Mesenchymal Progenitors Mitigates Age-Related Muscle Mass Loss and Neuromuscular Junction Degeneration
Source: Int J Mol Sci. 2021 Sep 23;22(19):10246. doi: 10.3390/ijms221910246 (PMC8549698; doi:10.3390/ijms221910246)
Supplement: Supplementary file 1 [file ijms-22-10246-s001.zip › ijms-1345412-SM.pdf]

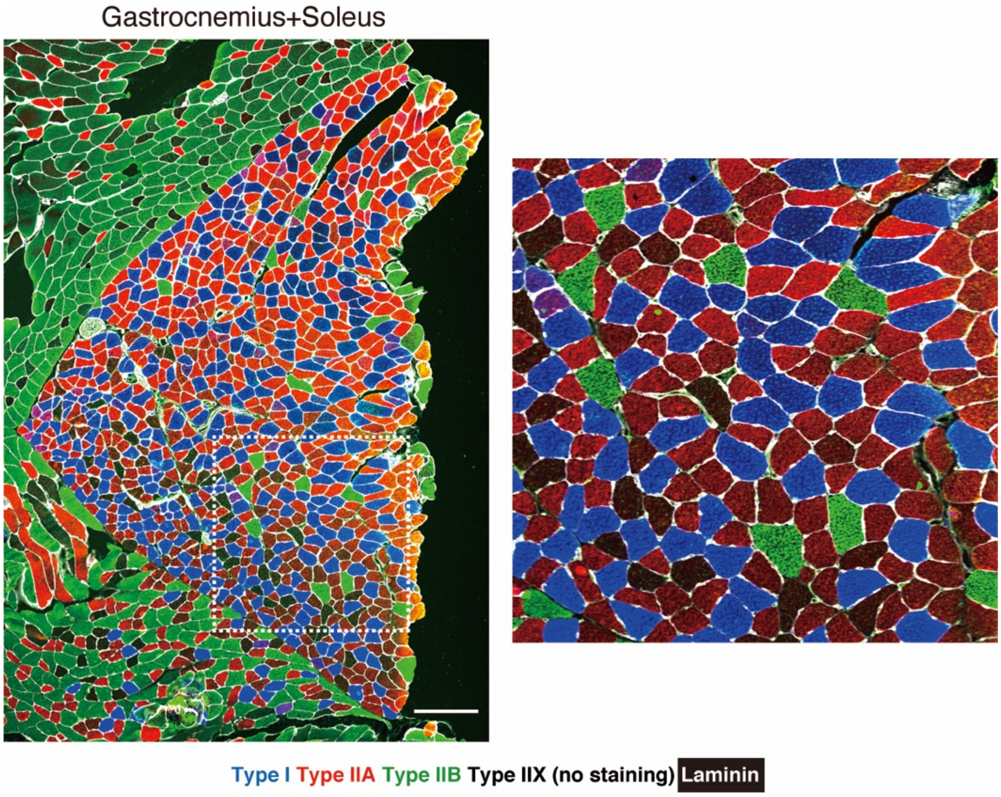

Figure S1. Detection of four types of myofibers.

Gastrocnemius and soleus muscle section of young mouse was stained with antibodies against MyHC I (blue), MyHC IIA (red), MyHC IIB (green), and laminin (white). The right panel are magnified views of boxed regions in the left panel. Myofibers negative for MyHC I, MyHC IIA, and MyHC IIB were considered as type IIX myofibers (no staining). Scale bar: 200 μm.
